# Bridging the gap: Phage manufacturing processes from laboratory to agri-food industry

**DOI:** 10.1016/j.virusres.2025.199537

**Published:** 2025-01-31

**Authors:** Elham Mohammadi, Mohammadreza Rahimian, Bahman Panahi

**Affiliations:** a NanoSciTec GmbH, Hermann Weinhauser str. 67, Munich 81867, Germany; b Department of Biology, Faculty of Basic Sciences, University of Maragheh, Maragheh, Iran; c Department of Genomics, Branch for Northwest & West Region, Agricultural Biotechnology Research Institute of Iran (ABRII), Agricultural Research, Education and Extension Organization (AREEO), Tabriz, Iran

**Keywords:** Bacteriophages, Phage manufacturing, Food safety, Antimicrobial resistance, Downstream processing, Upstream processing, Biocontrol, Agriculture biocontrol

## Abstract

Interest in bacteriophages (phages) as sustainable biocontrol agents in the agri-food industry has increased because of growing worries about food safety and antimicrobial resistance (AMR). The phage manufacturing process is examined in this review, with particular attention paid to the crucial upstream and downstream processes needed for large-scale production. Achieving large phage yields requires upstream procedures, including fermentation and phage amplification. In the meantime, downstream procedures, including purification, endotoxin removal, and formulation, is essential for guaranteeing product quality and regulatory compliance. Despite advances in upstream and downstream process optimization of phage production processes, these methods are not effectively utilized in manufacturing processes. Additionally, the commercialization of phage products is hindered by fragmented rules and inconsistent regulations. Emerging technologies such as enhanced chromatography, continuous processing, and encapsulating techniques provide prospects for increased stability, efficiency, and scalability to fill these gaps. Furthermore, by facilitating real-time process optimization, predictive quality control (QC), and unique phage product creation, the integration of artificial intelligence (AI) and machine learning has the potential to transform the phage manufacturing industry completely. In order to provide consistent standards, encourage innovation, and bridge the gap between academic research and commercial applications, this review identifies gaps and highlights the necessity of cooperation between academia, industry, and regulatory agencies. To effectively utilize phages' potential to improve food safety, fight AMR, and promote sustainable agricultural practices, the agri-food industry must advance phage manufacturing techniques and harmonize regulatory frameworks.

## Highlights

• Despite advances in upstream and downstream process optimization of phage production processes, these methods are not effectively utilized in manufacturing processes.
• By facilitating real-time process optimization, predictive quality control (QC), and unique phage product creation, the integration of artificial intelligence (AI) and machine learning has the potential to transform the phage manufacturing industry completely.
• To effectively utilize phages' potential to improve food safety, fight AMR, and promote sustainable agricultural practices, the agri-food industry must advance phage manufacturing techniques and harmonize regulatory frameworks.

## Graphical abstract

**Figure:**
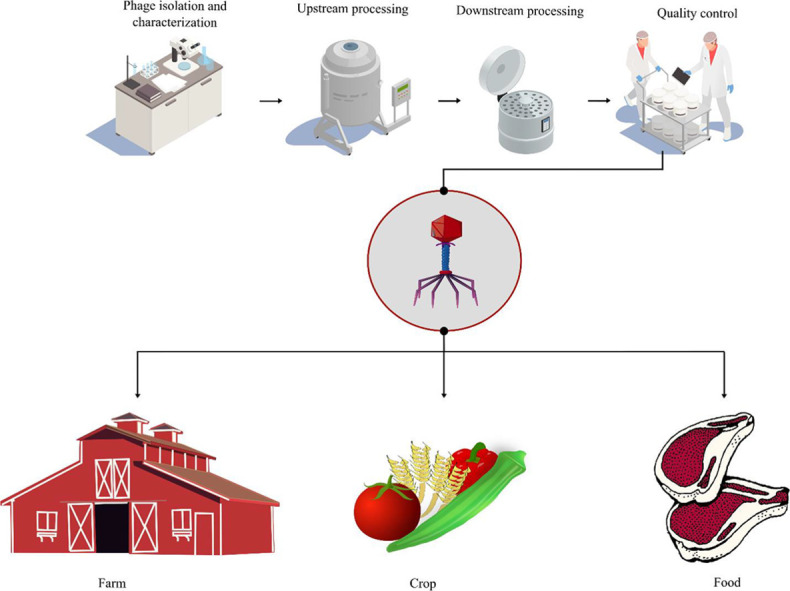

## 1 Introduction

Ensuring food safety and quality is a significant concern for the agri-food sector, especially when fighting bacterial infections that can jeopardize crop production and human health. Bacteriophages, or simply phages, have become a viable remedy since they provide a specific approach to controlling microorganisms. These viruses are an efficient biocontrol tool throughout the whole food production process, from farm to fork, since they precisely infect and eliminate bacteria (Imran et al., 2023).

Phages are viruses that reside in nature and are designed to attack bacterial cells. Due to their high prevalence in the ecosystem, isolating these viruses does not provide significant hurdles. Also, phages can target pathogenic bacteria without affecting non-target microorganisms, unlike broad-spectrum antibiotics that can target beneficial microbial populations. Moreover, the potential of phages to combine and show synergistic activity with other antibacterial agents such as enzybiotics and antimicrobial peptides such as nisin makes these viruses valuable biocontrol agents. Furthermore, phages' low stability in extreme environmental conditions, considered one of their main disadvantages, can be significantly avoided by encapsulating them into nanocarriers (Rahimian et al., 2024).

Since the mentioned advantages of phages and the emergence of antibiotic resistance among bacterial pathogens due to the discriminatory use of antibiotics in the food and agriculture sector (Cerqueira et al., 2019), phages have gained researchers' interest in using them instead of antibiotics in the agri-food industry. Additionally, various phages were isolated and characterized against main food spoilage bacteria such as *Escherichia coli* (Karami et al., 2024; Shin et al., 2024), *Salmonella* spp. (Sun et al., 2024; Wang et al., 2024b), *Yersinia* spp. (Hammerl et al., 2022; Kilgore et al., 2024), *Listeria monocytogenes* (Byun et al., 2024; Everhart et al., 2025), and *Campylobacter jejuni* (Henz et al., 2024; Zhang et al., 2024c), as well as significant infectious bacteria in agriculture such as *Pseudomonas syringae* (Liu et al., 2024b; Selcuk et al., 2024), *Ralstonia solanacearum* (Sasaki et al., 2024; Zhang et al., 2024b), *Agrobacterium tumefaciens* (Fortuna et al., 2023; Ramadhan et al., 2022), *Xanthomonas* spp. (Greer et al., 2024; Tarakanov et al., 2024), *Erwinia amylovora* (Besarab et al., 2024; Kim et al., 2024), and *Xylella fastidiosa* (Clavijo-Coppens et al., 2021; Sabri et al., 2024), which emphasize the high antibacterial potential of phages in agri-food biocontrol.

**Fig. 1:**
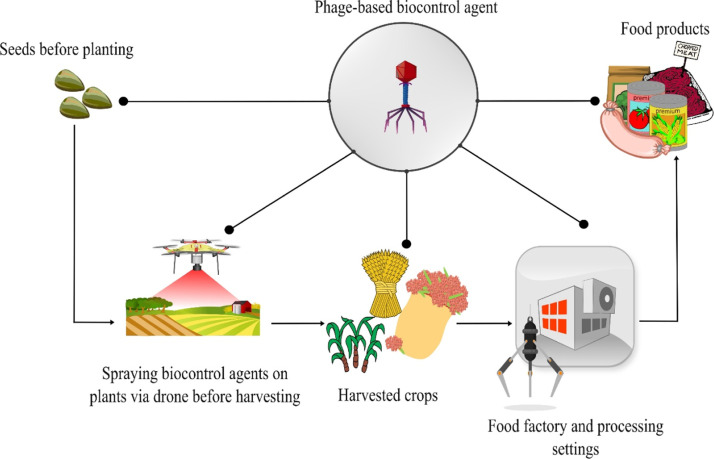
Application of phages as biocontrol agents in the agri-food industry, from farm to fork.

As illustrated in Fig. 1, phages can be applied at multiple points in the agri-food production chain. They can be applied to crops before harvest to lower disease burdens. Phages can also be applied directly to plants (Nga et al., 2021) or seeds (Erdrich et al., 2024) to help prevent diseases that threaten plant health or growth. Applying phages directly on the crops at the post-harvesting point can protect crops from spoilage (Zhang et al., 2024a). Also, phages are used in food processing facilities to remove contaminants from food products by spraying them onto surfaces or directly onto them. This use improves food safety and maintains the food's organoleptic qualities (Tan et al., 2024; Wang et al., 2024a). In addition, phages can also be used to sanitize surfaces and equipment in food processing settings to help avoid cross-contamination during production (Chen et al., 2022). While the potential of phages as a biocontrol agent against pathogenic bacteria in the agri-food industry is considerable, large-scale manufacturing of phages is demanded to provide the proper amount of phages to combat bacterial contamination.

It is necessary to consider the significance of producing phages on an industrial scale, particularly regarding the increasing demand for sustainable, safe, and efficient biocontrol strategies for food processing and agriculture. Phage manufacturing is crucial for producing high-quality, reliable phage formulations that may be used to control bacterial infections from farm to fork throughout the agri-food industry. Large-scale production makes the delivery of adequate phage amounts to satisfy practical applications possible, guaranteeing that phage products are stable, effective, and available in the required volumes (João et al., 2021). Upstream and downstream processing are the two primary operations of phage production. Cultivating bacterial host cells and carefully infecting them with phages are examples of upstream processing. Factors like temperature, pH, and nutrition availability must be optimized to optimize phage production at this stage. After that, downstream processing prioritizes concentrating and purifying the phage particles by removing them from contaminants and bacterial debris. Methods like filtration, centrifugation, and chromatography are frequently used to produce a pure phage product that satisfies quality and safety requirements. Phage production is essential in bringing phage applications to market since these steps guarantee phage-based biocontrol agents' scalability, safety, and efficacy (João et al., 2021).

Focusing on their function as biocontrol agents against bacterial diseases, this paper thoroughly analyzes the production process for phages utilized in the agri-food sector. It examines the crucial phases in phage production, starting with upstream procedures that optimize bacterial host cultures and phage infection parameters to maximum productivity. After that, the paper discusses downstream processing strategies, emphasizing formulation and purification processes that guarantee stable, high-quality phage products appropriate for use in food and agriculture. Also, important quality control (QC) procedures are covered to meet industry standards. In the end, phage productions in the agri-food sector are discussed, areas of the gap between academia and industry are examined and a future perspective is provided. This review aims to help the effective use of advances and laboratory optimizations in production processes and integrate phage technology into sustainable agriculture and food safety practices by offering insights into each step of phage manufacturing.

## 2 Upstream processing of phage manufacturing

Biological processes are crucial in biotechnology advancements that produce the desired product through living organisms such as cells. They include various steps that help optimize and produce the desired products. The bioprocess generally begins with an upstream process that includes phage and host selection, host propagation, and phage amplification using different feeding and processing strategies (Regulski et al., 2021). To identify the optimal conditions for producing bioproducts, bioprocessing is usually started on a small scale to optimize parameters and then scale up (Kensy et al., 2009). Following the upstream process, downstream processing is done to harvest the product and remove impurities; in this context, attention to bio-based products' safety and quality standards is significant. The cost of making a product in the upstream processes mainly includes the cost of raw materials and the implementation of bioreactors. In the downstream processes, the cost of purification and formulation directly affects the product's final cost (Sharma et al., 2022).

In the industry, automatically stirred tank bioreactors are mainly used for phage fermentation, which provides more optimal conditions for growth and production by controlling the parameters. When transferring from Erlenmeyer to the bioreactor, factors such as agitation rate and oxygen level and the amount of shear damage that affect the titer and health of the cells will have different conditions. For example, too little oxygen prevents the growth of host cells, and mechanical stress caused by stimulation can be harmful to cells that cannot withstand high shear stress, so it is necessary to conduct several studies to increase the efficiency and scale of the process (Malik et al., 2017). Bioreactors with automatic control of parameters can reduce diversity and deviation between cultures and increase the performance of phage culture, which is effective in preparing phage with high titer and low endotoxin (Luong et al., 2020). By performing a single culture in the bioreactor, instead of performing several shaking flask cultures, it is possible to produce more phage and optimally control the conditions in each batch, which not only reduces the risk of contamination but also reduces costs and allows the scaling of the process (Ali, 2020).

While the use of each bioreactor has certain advantages and disadvantages, the final type of bioreactor selected is generally based on the product and scale of the process. Investigating the principles of geometric similarity and hydrodynamic effect on culture and thermofluidics is essential when scaling a biological process in any reactor. Stainless steel bioreactors can be cleaned and sterilized despite high maintenance costs and limitations in transfer between cultures. They are suitable for large-scale microbial processes and can be used for a long time due to their high durability and stability. Single-use bioreactors, commonly used in laboratory research and development, require no time for cleaning and minimize the risk of contamination due to their single use. However, in addition to the high fixed cost due to less efficient oxygen mixing, they may need to perform better on large scales (Ali, 2020).

Typically, the process of cGMP production begins with fermentation of the host organism followed by phage infection of the culture, which on an industrial scale is usually performed using stirred tank bioreactors that may operate in batch, fed-batch, or continuous mode (Agboluaje and Sauvageau, 2018; Nabergoj et al., 2018). Optimal operating and infection conditions to maximize phage titer are often performed in separate bioreactors. Phage cocktails are usually produced independently and finally mixed before use, as different phages require different times, culture media and hosts for propagation (Chan et al., 2013).

In batch culture, the most common mode of operation in phage production is after the host cells have grown to a certain density and are infected at a specific initial MOI. At the same time as the bacteria grow, the phages also multiply, and finally, the cells are lysed (Santos et al., 2014; Sochocka et al., 2015). Although the batch operation is easy to control and produces high phage titers, low throughput, the need for preparation time before each run, labour, and large equipment volume are some of their limitations. Producing larger batches of a product in one reactor reduces the need to run multiple batches, which avoids the risk of contamination and differences in final product characteristics or performance and significantly reduces costs (João et al., 2021).

Fedbatch cultivation, which allows fresh nutrients to be added to the culture to improve the process's efficiency, is carried out in a 2-stage self-cycle in the bioreactor (Lee et al., 2021; Sauvageau and Cooper, 2010). In the first reactor, the cells grow until reaching the beginning of the stationary growth phase. Then, half of the bioreactor's content is transferred to the second reactor for phage infection. The remaining medium is immediately replaced with a fresh medium to start a new growth cycle (Krysiak-Baltyn et al., 2018a). This mode combines the advantages of batch and continuous processes by producing high phage titers, high volume throughput, reducing downtime at each production time, and significantly reducing the possibility of co-evolution by removing all bacterial cells between infection cycles (João et al., 2021).

In continuous cultivation, while the medium consumed by cells and phages is removed, the fresh culture medium is continuously supplied to the bioreactor (Jurač et al., 2019; Mancuso et al., 2018; Nabergoj et al., 2018). To prevent the occurrence of bacteriophage resistance or the mutation of strains during long periods of work, instead of single-stage continuous bioreactors (such as chemostat and turbidostat), multi-stage continuous processes (cellstat) are used (Husimi et al., 1982). In this continuous approach of several interconnected bioreactors, host cell growth and phage infection occur in separate bioreactors. Despite the complexities of setting up the continuous process of phage production, high productivity, and the possibility of significantly increasing the phage titer, they can significantly reduce the cost of production in terms of the size of bioreactors to the extent that it allows the use of single-use systems (Malik, 2021). Mancuso et al. also used a new reactor design consisting of 3 separate stages in the upstream stage of continuous phage production, which, by providing the possibility of controlling the physiological state of the bacterial population, significantly increased the overall efficiency of the phage process (Mancuso et al., 2018). With more understanding of the batch and fed-batch processes of phage production, it is likely that in the future, when there will be greater demand for phage products, continuous biological processes will be used that are more scalable and efficient by reducing the process footprint and production costs.

## 3 Downstream processing of phage manufacturing

Downstream processing includes all unit operations required to purify the phages to meet the bulk product specifications at regulatory levels. Usually, the most critical impurities are removed first, and the most difficult or expensive separations are left for the final stages of purification (Ali, 2020). Performing several single operations in the manufacturing process to clarify, concentrate, purify, polish, and formulate target phages to a certified purity is the overall goal of downstream processing, which should include a limited number of efficient and short steps to reduce complexity and minimize processing costs and residence time (Regulski et al., 2021). The culture broth produced at the production stage usually contains bacterial DNA, cell debris, LPS, environmental components, bacterial metabolites, and relatively low concentrations of phages, which can vary in viral preparations, so effective methods for removing bacterial debris likely remain the most significant challenge (Gill and Hyman, 2010). Unit operations in the design and operation of a biological process should be selected and arranged by considering the shape and size of phages, specific molecular interactions with adsorbent matrices, and the operational capacity of the units.

After the phage replication step, the first step in the downstream processing of viral particles is usually clarification by centrifugation or/and filtration (micro/depth), which provides the removal of cellular debris from the phage-rich medium (Bourdin et al., 2014; Liu et al., 2012; Rebula et al., 2023). Continuous low-speed centrifugation eliminates the need to use unsafe purification techniques such as cesium chloride (CsCl) gradient ultracentrifugation (João et al., 2021). Although microfiltration (with 0.22 μm membranes) is used after centrifugation to effectively remove impurities, phage loss and membrane fouling during filtration are potential bottlenecks that affect filtered phage titer and batch production time (Malik et al., 2023).

The second step in the downstream processing plan is the initial recovery of phages from cell lysates with the aim of volume reduction. Single operations such as ultrafiltration, diafiltration, sedimentation and aqueous two-phase extraction can concentrate the phage flow (Kramberger et al., 2015). The most commonly used filtration is tangential flow filtration (TFF) in concentration mode, where the phage lysate solution is circulated tangentially across the membrane surface under pressure and can be combined with diafiltration (DF) for buffer exchange (Malik et al., 2023). Although TFF is efficient and scalable in concentrating, removing low-molecular-weight impurities, and performing buffer exchange, crude bacteriophage preparations can damage the filter membrane rapidly, significantly limiting the membrane's life cycle (Rembhotkar and Khatri, 1989; Schwartz and Seeley, 2014). Another simple and cost-effective unit operation that can be used in this step is polyethylene glycol precipitation and subsequent ultracentrifugation with a CsCl gradient (Scopes, 1993; Yamamoto et al., 1970). In this technique, which helps to remove low molecular weight impurities, adding agents such as polyethylene glycol (PEG) and salts effectively remove phages from the solvent and provide high recovery (Branston et al., 2012). Cesium chloride gradient ultracentrifugation is an expensive and time-consuming technique. Regulatory agencies prohibit it due to the hazardous nature of CsCl or the use of Triton-X-100 as a surfactant, whose removal in the phage production process requires additional purification steps and reduces the overall efficiency of the process (Roshankhah et al., 2023). Aqueous two-phase systems (ATPS) consist of two water-soluble polymers or one polymer and one salt and are considered as another method of concentration of phages. In these systems, the components are divided into two immiscible aqueous phases under the influence of physical and chemical properties of phages and system parameters such as type and concentration of components, ionic strength and pH. In some systems, the high concentration of phase components may lead to the deposition of phages at the interface, which can have a negative effect on phage infection (Gomes et al., 2009).

Chromatography is a well-known option in downstream purification due to its scalability and robustness, which allows overcoming the challenges of phage purification by preparing samples of very high purity (Smrekar et al., 2011). In downstream processing, depending on the final product's purity requirements, the chromatography section can be performed in different interaction modes, such as size exclusion chromatography, ion exchange, and affinity chromatography in one or more steps (Kramberger et al., 2015). Due to the anionic nature of most phages, the most widely used chromatographic mode for the virus adsorption step in downstream processing on an industrial scale is to separate viruses from impurities based on the difference in their surface charge distribution (Kramberger et al., 2015). In ion exchange chromatography (AEC), partially purified phage solutions of high ionic strength are loaded onto AEC columns to release phages while removing bound impurities (Kramberger et al., 2015). Although AEC can produce purity comparable to CsCl density gradient methods and requires expensive equipment, AEC is a faster method with a scale-up advantage (Adriaenssens et al., 2012). Size exclusion chromatography (SEC) has effectively removed resistant proteins and low molecular weight impurities without significantly affecting phage viability. However, endotoxins are often associated with phages because they tend to form larger structures (Zakharova et al., 2005). Affinity chromatography targets impurities using specific ligands. Despite the wide range of settings and selections of the affinity chromatography purification device, this method is not ideal for high-throughput processes due to high consumption, low efficiency, and a significant reduction in phage titer in most cases (Luong et al., 2020). CaptoTM Core 700 beads use size removal and adsorption properties that combine principles to increase purification efficiency (James et al., 2016). This versatility in multimode chromatography shows the importance of using appropriate purification strategies for various phages with different structures and behaviours. However, traditional chromatographic media are not designed and optimized for purifying large particles such as viruses.

In contrast, new-generation chromatographic media, with membranes and monoliths such as CIM integrated columns, compared to particle-based resin and/or membrane, are used for efficient purification of various viruses (Kramberger et al., 2015). An efficient two-step chromatographic purification method using convective interaction media integrals (CIM®), in the first step, leads to 100 % bacteriophage recovery and in the second step leads to a significant reduction of endotoxin level (up to 7 logs) until reaching pharmaceutical grade purity (Rebula et al., 2023). Also, membrane chromatography, which is faster and more scalable, is a suitable alternative to resin-based chromatography, which has the limitation of binding to the external surface of resin particles. Recent studies have shown the effectiveness of using monoliths or membranes by providing better resolution and binding capacity for phage purification (Roshankhah et al., 2023). In general, the downstream chromatography process can be the most consistent approach for purifying phages on a large scale (Adriaenssens et al., 2012). In addition to high virus yield and efficient removal of impurities, the dynamic binding capacity (DBC) that determines the number of times required for purification is of great importance and significantly impacts process costs (Kramberger et al., 2015). Therefore, continuous research to select, develop, and modify these methods or their combination to increase the recovery and purity of phage is fundamental.

In the polishing stage in downstream processing, size exclusion chromatography (SEC) or UF/DF provides final impurity removal, excess concentration of an active substance (nanoparticles), and buffer exchange in the final formulation (Kramberger et al., 2015). Although size exclusion chromatography is effective in the simultaneous removal of impurities and buffer exchange, it has limitations in the flow rate of mobile phases and sample loading volume in each run (Gias et al., 2008). Ultrafiltration/diafiltration without SEC constraints can quickly increase virus concentration and buffer exchange but may require additional steps to remove bioburden. The sterile filtration step is essential before the final filling and finishing process for the final removal of biological load from the UF concentrate or SEC wash liquor (Malik et al., 2023). Although the sequence of operations of the selected unit in the production process is different depending on what industry the product is used for and with what purity level, it should be done with high efficiency and with minimum time and cost. João et al. reviewed the general downstream processing of phages in 4 different designs in detail (João et al., 2021).

Formulation and aseptic filling of containers is the last stage of downstream processing. Recent advances in phage encapsulation approaches improve its storage stability against environmental stresses and, especially in clinical applications, enable targeted delivery and controlled phage release at the site (Malik et al., 2017). Liquid and lyophilized formulations currently constitute the most common phage dosage forms. Phage delivery via liquid formulation is easy but can limit product durability, and large volumes of liquid phages are not easily transported (Jończyk et al., 2011). Concentrated and high-quality phage stocks can be freeze-dried or spray-dried to produce a concentrate or powder formulation that reduces molecular mobility and degradation kinetics (Vandenheuvel et al., 2014; Vinner et al., 2019). The use of excipients such as trehalose, sucrose and mannitol sugars before drying protects phages against dehydration and thermal stress, which helps to improve their shelf life (Zhang et al., 2020). Although it is scalable and has a good application in the industry, drying phages by spray and freezing still needs more optimizations to prevent phage titer reduction. Optimizing phage drying with a spray dryer for mixing with animal feed is one of the few studies published commercially (Thanki et al., 2022).

Since phages are inherently variable in composition, stability, and biological activity, at the end of production, as with any other product, QC is critical throughout product development from laboratory scale to commercialization. QC in terms of shelf life, physicochemical and biological stability and the degree of freedom from impurities in accordance with the approved specifications is essential not only in the final product but also in the different stages of the production process because sometimes even minor changes in the upstream processing can lead to a less efficient downstream processing (Kramberger et al., 2015). QC is essential during the manufacturing process of phage-based products to ensure the safety, efficacy, and stability of these cutting-edge solutions across various industries. The rules and guidelines that control these procedures are intended to reduce the risks of contamination, variations in product potency, and unintended phage application consequences. These regulations guarantee that the products fulfil the requirements of the industry in which they are meant to be used (Mutti and Corsini, 2019).

In general, the FDA and other regulatory agencies emphasize how crucial it is to manage both the production process and the quality of the final product. For every batch produced, this includes strict testing procedures to verify conformity to predetermined standards (Almeter et al., 2022). In phage manufacturing, batch-to-batch consistency is essential for preserving each batch's purity, potency, and efficacy, particularly in therapeutic applications and biocontrol in food safety. This requires standardized production procedures that consistently yield consistent phage preparations, stringent testing procedures to confirm the identity and activity of phages generated in various batches, and resolving issues with stability and efficacy over time (Bretaudeau et al., 2020; Malik et al., 2023).

Table 1 provides a helpful summary of research on phage manufacturing. Comparing these investigations shows significant differences in methods, phage titers attained, and integration (or lack thereof) with downstream processes. The understudied downstream processing of phages, including purification, concentration, and formulation, highlights a crucial gap in phage manufacturing research, whereas upstream optimization has received the most attention. A key parameter often reported in the studies is the phage titer, which measures the efficiency of upstream processes. The studies in the table reported a wide range of phage titers depending on the cultivation system (flask vs. bioreactor), host strain, and process conditions.

**Table 1:** Phage manufacturing academic studies.

Phage	Host	Upstream process	Downstream process	Phage final titer	Refs.
T4	*E. coli*	Bioreactors with a working volume of 1 L, Two-stage self-cycling system (semicontinuous)	–	7.59 × 10^14^ PFU/mol CO_2_	Sauvageau and Cooper (2010)
T7	*E. coli*	Bioreactors with a working volume of 3 L, multi-fermenter system (batch)	–	1.5 × 10^10^ PFU/mL	Smrekar et al. (2011)
T4	*E. coli*	Bioreactor with a working volume of 8 L, batch culture system	–	1.2 × 10^16^ PFU/mL	Sochocka et al. (2015)
T3	*E. coli*	Bioreactor with working volume of 1 L, multi-stage bioreactor including two continuous and third semi-batch system	–	2 × 10^11^ PFU/mL	Mancuso et al. (2018)
T4	*E. coli*	Bioreactor with a working volume of 1 L, cellstat system (continuous)	–	2.4 × 10^13^ PFU/day	Nabergoj et al. (2018)
T4 and K	*E. coli and S. aureus*	A 5 L stirred-tank bioreactor with a working volume of 3 L	Centrifugation and filtration	7.89 × 10^13^ PFU/ml for T4 and 1.03 × 10^13^ PFU/ml for phage K	Ali (2020), Ali et al. (2019)
Φ CS01	*Cronobacter sakazakii*	A 5 L bioreactor, Two-stage self-cycling system (semicontinuous)	Centrifuge, microfiltration and ultrafiltration	5 × 10^10^ PFU/mL	Lee et al. (2021)
PP-01	*Pseudomonas aeruginosa*	Erlenmeyer flasks with a working volume of 200 mL	Centrifugation with pore size filters, CIM monolithic chromatography, contains three different polishing columns (two-stage chromatography)	> 10^11^ and > 10^12^ PFU/ml*	Rebula et al. (2023)
T7	*E. coli*	Preparing cultures in volumes up to 5 mL, batch	0.45 μm pore size microfilter, anion exchange membrane chromatography system	NM	Roshankhah et al. (2023)
Phage75	*E. coli*	CellMaker bioreactor with a working volume of 3 L	–	∼ 3.16 × 10^9^ to 1.26 × 10^10^	Wiebe et al. (2024)

*: In this study, different phage yields higher than 10^11^ and 10^12^ PFU/ml were obtained from different chromatography columns. NM: Not mentioned

Phage propagation is fundamental in phage manufacturing, as evidenced by many upstream-focused studies. The examined investigations frequently concentrated only on increasing titers, ignoring the downstream processes. For instance, some bioreactor research (Nabergoj et al., 2018; Sochocka et al., 2015) produced significant titers without addressing the possible difficulties of downstream purification. The downstream aspect of phage manufacturing still needs to be explored, in contrast to the substantial study on upstream processes. Delivering top-notch phage preparations that satisfy regulatory requirements and are appropriate for end-use applications depends heavily on downstream procedures (Łącki et al., 2018). The lack of information in this field indicates that more research is required to address issues such as phage aggregation, endotoxin contamination, phage viability during storage, and the elimination of host cell debris. This gap in downstream process development hinders the large-scale commercialization of phage-based products. Guaranteeing phage formulations' safety, purity, and uniformity in the absence of downstream solid procedures is challenging. To satisfy the requirements of industrial production, the scalability of purifying techniques like ultrafiltration, chromatography, or precipitation procedures must also be assessed (Łącki et al., 2018). Since upstream and downstream processes still need to be integrated, more research is required to develop end-to-end manufacturing pipelines. Also, due to their well-established effectiveness against specific bacterial targets and well-characterized nature, most of the research in the table focuses on evaluating standard phages. Hence, evaluating the manufacturing process of novel isolated phages is still in demand.

## 4 Current status of phage products in the agri-food industry

Owing to the spreading and emergence of the antimicrobial resistance (AMR) crisis among pathogenic bacteria, several companies have withdrawn from new antibiotic research initiatives to concentrate their development and research resources on alternative therapeutic areas despite the pressing need for new antibacterial therapies (Rahimian and Panahi, 2024). Recent studies have extensively reviewed phage applications in the agri-food industry. They discuss the potential of phage products to combat AMR and enhance food safety across various stages of production, from crop protection to food processing​ (Fernández et al., 2018; Huang et al., 2022; Moye et al., 2018).

Significant developments in product commercialization, regulatory approval, and incorporation into current food safety procedures are seen in recent trends in phage products. Phage-based products that target pathogens, including *Salmonella* spp., *E. coli*, and L. *monocytogenes* in fresh produce and ready-to-eat foods, have received approval from regulatory agencies like the European Food Safety Authority (EFSA), U.S. food and drug administration (FDA) and U.S. environmental protection agency (EPA) in the U.S., Europe, and other countries. Due to such circumstances, companies have launched a range of phage solutions to target these infections while maintaining the food's beneficial microbiota (Table 2). High specificity, adaptability, and low environmental effect are some of the benefits of phages. They have further fueled their acceptability as a substitute or supplemental intervention to heat treatments and chemical sanitizers, frequently degrading food's nutritional value and sensory appeal (Fernández et al., 2018; Huang et al., 2022; Moye et al., 2018). In light of these advancements, this section will examine the current manufacturing procedures utilized in the production of phages, emphasizing scalable methods that satisfy industrial safety and effectiveness standards.

**Table 2:** The list of phage companies in the agri-food industry.

Company	Focus area	Products/services	Target bacteria	Website
COMbio (Malaysia)	Crop protection	Phage-based microbial pesticides targeting soft rot diseases in crops	Not mentioned specifically	www.combio-aimst.my
Intralytix, Inc. (USA)	Food safety and biocontrol	ListShield™	L. *monocytogenes*	www.intralytix.com
		SalmoFresh™	*Salmonella* spp.	
		ShigaShield™	*Shigella* spp.	
		EcoShield™ PX	*E. coli* O157:H7	
		CampyShield™	*Campylobacter* spp.	
		Ecolicide®	*E. coli* O157:H7	
		SalmoLyse®	*Salmonella* spp.	
		ListPhage™	L. *monocytogenes*	
		Ecolicide® PX	*E. coli*	
OmniLytics Inc. (USA)	Crop protection	AgriPhage™	Various types of bacteria	www.omnilytics.com/
	Food safety	Finalyse SAL	*Salmonella*	
		Finalyse STEC	Shiga toxin-producing *E. coli*	
		Finalyse O157	*E. coli* O157:H7	
Enviroinvest Ltd. (Hungary)	Crop protection	Erwiphage	*E. amylovora*	www.enviroinvest.hu/
APS Biocontrol Ltd. (Scotland)	Crop protection	Biolyse® BP	Soft rot *Enterobacteriaceae*	www.apsbiocontrol.com/
Micreos (Netherlands)	Food safety	PhageGuardE™	*E. coli*	www.micreos.com/
		PhageGuardS™	*Salmonella* spp.	
		PhageGuardListex™	L. *monocytogenes*	
		Salmonelex™	*Salmonella* spp.	
NexaBiome (UK)	Crop protection	agriPHIX™	Various types of bacteria	www.nexabiome.com/
FINK TEC GmbH (Germany)	Food safety	Secure ShieldE1	*E. coli*	www.finktec.com/
		Applied Phage Vegetable S2	*S. enterica*	
Phagelux (Canada)	Food safety	SalmoPro®	*Salmonella* spp.	www.phageluxbio.com/
Gum Products International (Canada)	Food safety	GPI Biotech VAM-S	*S. enterica*	www.gpiglobal.com/
SK8 Biotechnologies Inc. (Canada)	Food safety	SK8 VAM-S	*Salmonella* spp.	www.sk8biotech.com/
Qingdao Phagepharm Bio-Tech Co., Ltd (China)	Food safety	*Salmonella Enteritidis* Phage Preparation (Strain SP8)	*S. Enteritidis*	www.phageseeker.com/
UniFAHS (Thailand)	Food safety and biocontrol	PhagePrompt™	Various types of bacteria	www.UniFAHS.com

### 4.1 Manufacturing process of phages for food applications

**Fig. 2:**
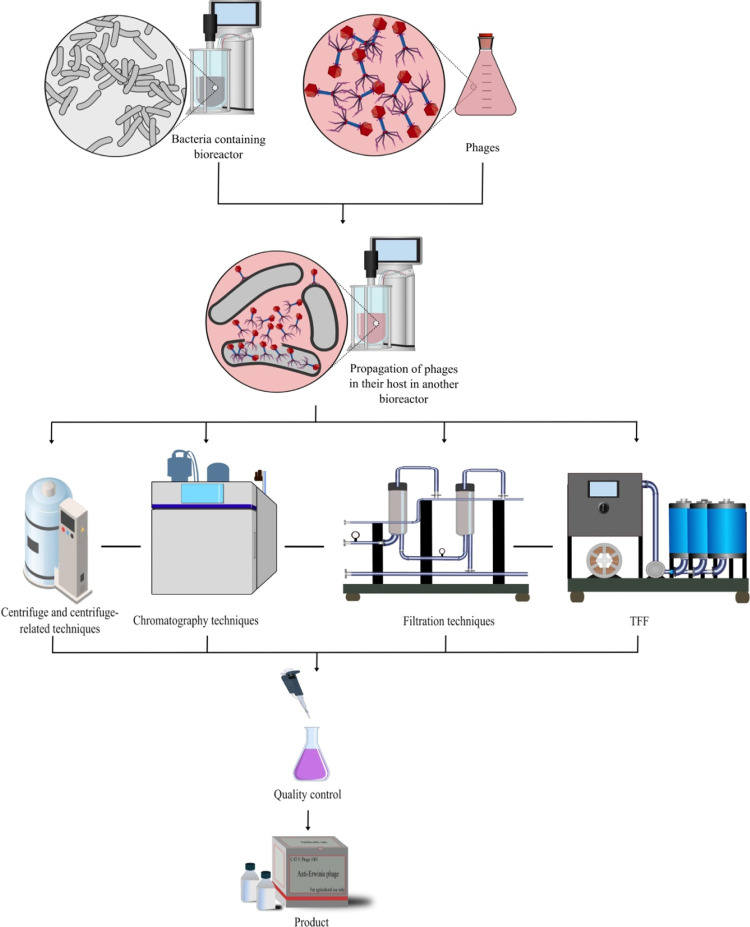
An overview of the phage manufacturing process.

As worries about antibiotic resistance and food safety increase, phages focus on controlling bacterial contamination in food production (Fernández et al., 2018; Huang et al., 2022; Moye et al., 2018). In this regard, several crucial processes in the phage manufacturing process guarantee the creation of high-quality phage products appropriate for various food applications (Fig. 2).

**Fig. 3:**
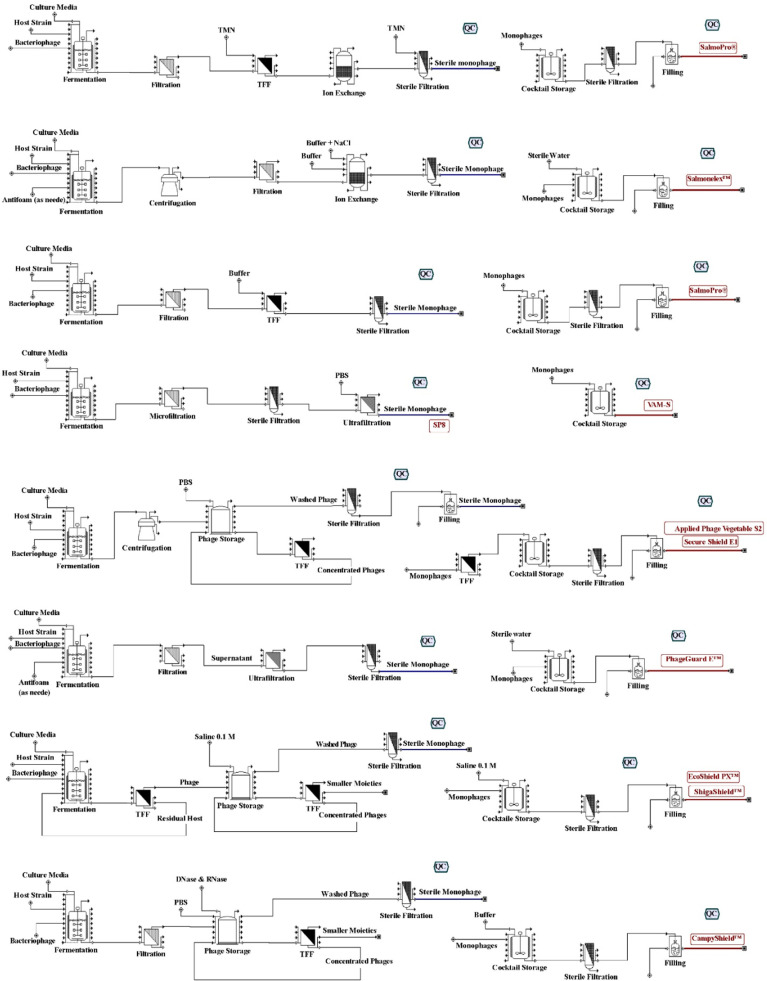
Workflow of phage manufacturing processes from obtained gras notices.

Table 3 and Fig. 3 were generated based on available and downloadable gras notices from the FDA database (https://www.fda.gov/search?s=GRAS%20notice%20phage&sort_bef_combine=rel_DESC&page=0). Sequential illustrations in Fig. 3 show every step of the production process, from the host strain preparation to the ultimate storage and packing of the phage-based formulations. The procedures are customized for individual products, considering differences in their composition and intended applications. According to the obtained gras notices, companies use bioreactor batch systems to propagate their phages. Despite the upstream process, utilized downstream processes in companies are varied. The clear marking of QC checkpoints guarantees compliance with safety and effectiveness requirements. The FDA's regulatory procedures guarantee the safety and efficacy of these phage formulations for consumers.

**Table 3:** Phage manufacturing industrial phage products.

Phage	Commercial name	Host	Upstream process	Downstream process	Phage final titer (PFU/mL)	Regulatory certification
BP-63 and BP-12	SalmoPro®	*E. coli*	Aerobic batch bioreactor system	Filtration, TFF, ion exchange chromatography, sterile filtration	10^9^	GRN* 603
FO1a and S16	Salmonelex™	*Salmonella bongori*	Aerobic bioreactor system	Centrifugation and filtration	10^11^	GRN 630
BP-63 and LVR16A	SalmoPro®	*E. coli* and *S. enterica*	Aerobic batch bioreactor system	Filtration, TFF, sterile filtration	10^10^	GRN 752
Three to eight phages	EcoShield PX™	*E. coli*	Aerobic batch bioreactor system	Centrifuge, TFF, sterile filtration	∼ 3 × 10^10^	GRN 834
Phi_16, Phi_78, and Phi_87	VAM-S	*Salmonella Typhimurium*	Aerobic bioreactor system	Micro-filtration, sterile filtration, and ultra-filtration	> 10^11^	GRN 917
J350, J375, J386	CampyShield™	*C. jejuni*	Aerobic batch bioreactor system	Filtration, TFF, sterile filtration	≥ 10^10^	GRN 966
11 phages	Applied Phage Vegetable S2	*S. bongori, Salmonella paratyphi* B and *E. coli*	Aerobic bioreactor system	Centrifuge, TFF, sterile filtration	> 5 × 10⁹	GRN 1070
Salmonella Enteritidis Phage Preparation (Strain SP8)	SP8	*S. Enteritidis*	Batch bioreactor system	Centrifuge, micro-filtration, sterile filtration, ultra-filtration	≥ 10^10^	GRN 1134
EP75 and EP335	PhageGuard E™	*E. coli*	Batch bioreactor system	Filtration, ultra-filtration, sterile filtration	≥ 2 × 10^11^	GRN 757
5 phages	ShigaShield™	*Shigella sonnei*	Aerobic batch bioreactor system	Centrifuge, TFF, sterile filtration	≥ 10^10^	GRN 672
12 phages	Secure Shield E1	*E. coli*	Aerobic bioreactor system	Centrifuge, TFF, sterile filtration	> 5 × 10⁹	GRN 724

*: Gras Notice. Each GRN is downloadable content that can be obtained from the FDA database.

To ensure their safety and effectiveness, phage products must adhere to strict QC procedures covering a range of factors. The FDA categorizes some phage products as generally recognized as safe (GRAS), making incorporating them into food products easier. Strict testing procedures that evaluate phage concentration, purity, and sterility are part of QC in this field (Bretaudeau et al., 2020). This is crucial to avoid adverse consumer effects, especially when phages are used in ready-to-eat foods. Another crucial factor is microbiological purity; sterility testing must verify that no microbial growth happens after a predetermined incubation period, usually longer than 14 days. This guarantees that dangerous microorganisms will not be added to food products by phage preparations (Würstle et al., 2024). According to the GRAS notice documents, batch-to-batch consistency ensures dependable performance and safety across various manufacturing cycles for any monophage and phage cocktail product (Bretaudeau et al., 2020; Sauvageau and Cooper, 2010). Several phage formulations suitable for food have been developed and successfully introduced. The approval of such formulations demonstrates the regulatory confidence in phage uses in the food business.

A Salmonella-specific phage cocktail called SalmoPro® is produced using the first method described in Fig. 3. The process starts with fermentation using host strains and culture media. Endotoxins are eliminated by ion exchange after the product has been clarified by post-fermentation, filtration, and TFF. Before the substance is finally packaged as a cocktail or stored as sterile monophages, sterile filtering guarantees microbial purity (GRN 603). With crucial processes like ion exchange and sterile filtration, the subsequent procedure, Salmonelex™, takes a similar path. In this case, the unique feature is the direct incorporation of sterile monophages into the cocktail, which is then diluted and kept at the application sites (GRN 630).

VAM-S, a bacteriophage cocktail targeting *Salmonella*, strongly emphasizes ultrafiltration during processing to further purify the phage preparation. The final cocktail is created by combining the sterile monophages and kept in a refrigerator (GRN 917). Centrifugation and TFF are integrated into the CampyShield™ process, which is intended to combat *Campylobacter*. This guarantees the elimination of bacterial debris and phage purification, after which the final formulation is blended and stored (GRN 966). Ultrafiltration is used in SP8 manufacture to concentrate the phage and eliminate any remaining host components. The procedure is designed to produce sterile monophages packaged in a form ready for use (GRN 1134).

Targeting *E. coli* O157, PhageGuard E™ uses a simple approach, including filtration, sterile filtration, and blending sterile monophages. Before being stored and used, the product is put through a rigorous QC process (GRN 757). EcoShield PX™ (GRN 834) and ShigaShield™ (GRN 672) highlight the creation of multi-phage cocktails using strict purification procedures, such as tangential flow filtration and sterile filtering. The concentrated versions of both products are diluted and stored before use. Secure Shield E1 (GRN 724) and Applied Phage Vegetable S2 (GRN 1070) demonstrate procedures that use host strains and phosphate-buffered saline (PBS) washing. By removing carryover impurities, these procedures guarantee that the phages fulfill the necessary purity and efficacy requirements.

Fig. 3 is an essential part of the article since it clearly shows how the various production and optimization techniques investigated in academic studies differ from the industrial procedures used to manufacture phages. Acting as a helpful road map, it illustrates how theoretical developments are applied to extensive, controlled production systems and draws attention to the substantial space for growth in the industry's efficient use of these developments. Even with the range of production methods, it emphasizes how most food sector phage product manufacturers consistently rely on single-stage bioreactors, batch processing, and filtration, especially of the TFF type. This comparison highlights the necessity for additional innovation and optimization to balance efficiency, scalability, and regulatory compliance. Ultimately, it offers a valuable resource for scientists and industry experts looking to create manufacturing procedures that combine the advantages of scholarly research with practical industrial uses, propelling the field toward more effective procedures and premium, reasonably priced bacteriophage products.

### 4.2 Phage manufacturing in the agriculture industry

In agriculture, phages have become a novel and promising answer, especially as biocontrol agents against plant diseases. Due to their high specificity in destroying bacterial pathogens while sparing beneficial microbes, they are positioned as a sustainable substitute for chemical pesticides and antibiotics (Sieiro et al., 2020). The increasing use of phage-based treatments in agriculture is evidenced by commercial products such as Enviroinvest's Erwiphage and OmniLytics' AgriPhage™. These products demonstrate how effective phages are in improving plant health, lowering reliance on chemical treatments, and combating the worldwide problem of antibiotic resistance.

In contrast to the relatively well-documented procedures in the food companies, more comprehensive literature needs to detail the production workflows unique to the agricultural sector, despite the proven effectiveness and expanding market for phage products in this field. This disparity makes establishing best practices for increasing production and guaranteeing quality compliance in agricultural applications more difficult. To protect the environment and public health, agricultural phage products must adhere to strict regulatory requirements, especially those established by the EPA.

For example, to ensure the safety, consistency, and effectiveness of microbial pesticides, including phages, the EPA has set strict rules under OPPTS 885.1200 to control their manufacturing. To guarantee that the product used in the field corresponds to the version evaluated for safety, these standards mandate that manufacturers thoroughly characterize their products by identifying phages down to the strain or serotype level. By putting robust QC processes in place, the production process must strictly limit chemical and biological contamination. These consist of biological, chemical, or serological testing to confirm product consistency and integrity across batches. Manufacturers must also explain and verify their procedures to guarantee the purity of the starting cultures, intermediate products, and final phage production (EPA, 1996).

Additionally, Acute pulmonary toxicity and pathogenicity testing for microbial pesticides, including phages, are included under the EPA's OPPTS 885.3150 criteria to guarantee their safety after pulmonary exposure (EPA, 1996). Environmental impact assessments are frequently required to ensure that phages do not disturb ecosystems, whereas safety testing concentrates on non-toxicity for plants, animals, and non-target organisms. Although purity standards exist, they are not as strict as those for pharmaceuticals. To ensure that phage preparations do not introduce dangerous bacteria into the agricultural environment, ensure that contaminating microorganisms are not present (Svircev et al., 2018). These assays are intended to give vital information about microbial pesticides' pathogenicity, toxicity, and infectivity when given intratracheal or intranasal routes in a single high-dose exposure. Clinical symptoms, death rates, and microbial clearance must be observed during the investigation. Infectivity and persistence must also be evaluated in tissues, organs, and body fluids. To ensure consistency in outcomes, the microbial pesticide used in testing must have the same form as the one that will be applied. According to the guidelines, phages that produce toxins must be closely monitored, and additional acute toxicity investigations must be conducted to detect and isolate hazardous components. The guidelines also stress ensuring that enumeration techniques are consistent and their sensitivity to identifying microbial reproduction is confirmed. By ensuring that microbial pesticides adhere to safety regulations, these stringent testing procedures reduce environmental and public health hazards (EPA, 1996). These strict guidelines highlight the need to continue using high-quality production techniques for agricultural phage products to guarantee their efficacy, safety, and regulatory compliance.

## 5 Current gaps and future perspective

Despite its potential for transforming biocontrol strategies against bacterial diseases, the phage manufacturing landscape, especially in the agri-food sector, confronts substantial gaps and hurdles. One of the essential aspects of phage products is producing phages in powder form instead of liquid form. According to previous studies, powder-form phages have longer shelf-life, are more stable during manufacturing, and can be consumed in multiple routes (Li et al., 2021; Zhang et al., 2021). However, while phage production in powder form has been evaluated in previous studies for veterinary medicine applications (Thanki et al., 2022), there is a lack of data on the agri-food industry.

Also, the regulatory structure, which needs to be more cohesive and balanced, is a significant cause for concern, especially concerning food and agricultural applications. In contrast to the medical field, which has well-defined standards, the agri-food sector does not have a single set of guidelines, which makes compliance difficult and frequently conflicting. Currently, good manufacturing practice (GMP) guidelines, which focus on ensuring safety, efficacy, and quality throughout the manufacturing process, are not as clear and transparent for phage products in the agri-food industry as they are for other products in this industry, which creates uncertainty in compliance and safety measures, and gaps in oversight (Fernández et al., 2018; Garvey, 2022). Acceptance of phage applications in food safety and environmental health requires the development of clear regulations and standardized GMP protocols that ensure effective implementation (Liu et al., 2024a). On the other hand, equipment design, use of effective purification methods, and training of personnel involved in the production process under GMP standards are essential to prevent contamination and maintain the integrity of the phage product (Garvey, 2022). For example, the FDA's GRAS regulations for food applications have different endotoxin level standards. While some GRAS notices prescribe much lower thresholds, like 25,000 EU/mL (GRN 834 and 603) or even 2500 EU/mL (GRN 917 and 1134), others set limits as high as 250,000 EU/mL for concentrated products (e.g., GRN 630 and an academic review article by Jaglan et al., 2022). Production and QC procedures are made more difficult by the difference.

Furthermore, although it guarantees safety, the FDA-mandated phage concentration cap of 10⁸ PFU/g in food may unintentionally reduce the effectiveness of phage formulations. Manufacturers have responded by creating concentrated phage solutions that need to be diluted before application, such as Phagelux's SalmoPro®. However, this approach raises the question of whether such strict limitations are required for safety without sacrificing the efficacy of biocontrol agents.

The difficulties are considerably more noticeable in agriculture. There are few regulatory frameworks in this field despite data demonstrating the effectiveness of phage-based therapies. The lack of thorough guidelines for phage titer requirements, safety limitations, and production techniques hinders the broad use of phages in crop protection. According to studies like Holtappels et al. (2021), study phage titers between 10⁶ PFU/mL to 10¹¹ PFU/mL are required for efficient biocontrol in crop production. However, it would be difficult to achieve a sufficient bacterial reduction in agriculture if strict food-grade standards were used. Setting context-specific guidelines is essential to balance safety and effectiveness in various applications.

The other essential aspect of phage manufacturing is that it faces regulatory issues in prophage contamination in the final product. Prophage presence in the final product is not desirable due to their potential to spread antibiotic resistance genes (Wang et al., 2020) and virulence factors among bacteria (Castillo et al., 2018) and stimulate toxin generation in specific bacteria (Sekulovic et al., 2011). Hence, prophage contamination in the final phage-based product cannot be allowed. Host bacteria must be scrutinized for the presence of prophages in their genome, and penetration of lysogenic phages into batches must be inhibited in order to have a prophage-free final product (Jérôme, 2020). However, the lack of rigorous regulations and standards in the case of prophage contamination is another hurdle to phage manufacturing. While few agencies set some regulations for prophage contamination in phage-based therapeutics (Jérôme, 2020), there is also a need to have such rules for the agri-food application of phages to avoid any calamity.

Another significant drawback is the need for comparability and integration in phage manufacturing research. Current research frequently overlooks downstream concerns, including purification, formulation, and endotoxin removal, in favour of concentrating primarily on improving upstream procedures to increase phage titers. Studies comparing manufacturing methods for the same phage type (such as T4) show this imbalance, as variations in reported phage concentrations are exacerbated by using non-standardized units, which precludes cross-study comparisons. A thorough analysis comparing batch, fed-batch, and continuous manufacturing modes under standardized conditions is desperately needed to determine the best strategies for scalability, efficiency, and regulatory compliance.

Continuous processes offer high scalability and throughput compared to batch processes, reduce product quality variability, and are more cost-effective (Nabergoj et al., 2018). However, in larger-scale phage production processes, batch mode is often preferred due to the convenience and possibility of greater process control and reduced risk of contamination (João et al., 2021). However, one of the crucial factors affecting the process mode is the dynamics of the phage host, which must be carefully controlled to prevent the development of resistance and mutation, especially since it is more challenging to manage in continuous systems (Mancuso et al., 2018). Batch systems reduce the risk of contamination by limiting production to discrete cycles (Pirnay et al., 2018). Continuous mode seems more suitable for phages with fast replication and stable dynamics that experience less stress or productivity loss over time. In these conditions, where the host and phage constantly interact for a long time, continuous production is possible without significant interruptions (Miller et al., 2020). However, to reduce the possibility of resistance or mutation and improve efficiency in continuous mode, it is proposed to use multi-stage systems in upstream processing that manage separate stages of host cell growth and phage infection (Mancuso et al., 2018). Although optimizing such systems has recently received more attention in academic centers, industries still adhere to single-stage and batch systems. To overcome these obstacles, future research should focus on developing hybrid systems that integrate the safety and control of batch processes with the efficiency of continuous methods to improve the efficiency and reliability of industrial phage production.

Despite the diversity of methods and operations in downstream phage processing, there is room for further advancements to address the technological gaps between academic research and industrial practices. While filtration methods, especially TFF, are widely used in phage production in industry, they have significant limitations, especially in filter fouling and pressure drop issues that require precise flow control (Isu et al., 2022; Romann et al., 2024). On the other hand, TFF requires complementary purification methods to effectively remove endotoxins and achieve the high purity levels required for specific applications to increase scalability and efficiency (Hietala et al., 2019). Academic studies using advanced chromatography techniques, such as multimodal chromatography and monolithic columns, have shown superior separation efficiency. However, these methods require further optimization to become cost-effective and scalable for various industrial applications (Rebula et al., 2023; Roshankhah et al., 2023). Additionally, encapsulation methods such as freeze-drying and spray-drying can stabilize phages and enhance delivery. However, further studies are required to minimize phage titer loss and ensure robustness under industrial production conditions (Malik et al., 2017). To fill this gap, optimizing these technologies and effectively integrating them into industrial-scale processes can ensure the production of high-quality and cost-effective phage products for broader applications.

Mathematical (computational) modeling is one of the well-known applications that can be utilized to better understand phage-bacteria interactions during the phage manufacturing process. This type of modeling, which was well-reviewed and described in previous studies (García et al., 2019; Styles et al., 2021), was used for all three phage manufacturing modes. When mathematical modeling was developed for academic applications, even in batch mode (Beretta and Kuang, 1998; Santos et al., 2014), we couldn't find any evidence proving mathematical modeling utilization in industry. Also, mathematical modeling can be a helpful method to optimize the phage manufacturing process when the growth parameters of bacteria and infection parameters of phages (e.g., adsorption dynamics, latent period, and burst size) are obtained accurately (Krysiak-Baltyn et al., 2018b). The necessity of achieving such data emphasizes developing standard methods to calculate phage infection parameters. One-step growth experiments serve as a valuable method for achieving the latent period and burst size of phages, and they are also used in phage manufacturing modeling (Santos et al., 2014). However, the lack of standard protocol to assess this experiment and consequently calculate the burst size of phages may have a negative impact on the accuracy of the modeling. Considering latent period and burst size as parameters that have high impacts on the phage manufacturing process (Beke et al., 2016; Santos et al., 2014), phage experts must agree on the best way to perform a one-step growth experiment and an accurate single formula to calculate the burst size of phages.

Machine learning and artificial intelligence (AI)-based tools have been shown to provide great opportunities to increase the pace and accuracy of phage studies (Nami et al., 2021). These technologies also have the potential to revolutionize the phage manufacturing industry. By processing extensive datasets, AI can predict host-phage dynamics (Rahimian et al., 2024), improve bioreactor conditions, and detect possible production bottlenecks (Cantarero-Rivera et al., 2024). Moreover, AI can potentially improve QC (Mahmood et al., 2024), accelerate purification procedures (Shahouni et al., 2024), and predict phage stability in various storage scenarios (Mallick et al., 2024).

However, it is noteworthy that the production parameters for each phage may vary significantly, adding complexity to the preparation process. Thus, applying AI and machine learning techniques in this context requires the processing of large and diverse datasets to accommodate these variations effectively. Integrating AI with whole-genome sequencing data can provide deeper insights into phage-host interactions (Rahimian and Panahi, 2024) creating highly effective phage cocktails tailored to specific bacterial targets (Keith et al., 2024).

Incorporating Industry 4.0 approaches, such as integrating AI with process analytical technology (PAT) and machine learning models, can optimize phage production processes. This integration enables real-time monitoring and adaptive control of critical parameters, enhancing scalability and efficiency (Narayanan et al., 2020). Additionally, these digital tools facilitate scaling production from pilot to industrial levels while adhering to regulatory standards, contributing to the advancement of Bioindustry 4.0 (Rathore et al., 2023).

The agri-food sector must prioritize creating thorough and uniform regulatory requirements to guarantee the safety and effectiveness of phage products while promoting innovation. Cooperation between academia, industry, and regulatory agencies is crucial to closing the gaps in research and practice. Scaling up phage production will require moving toward end-to-end studies that combine upstream and downstream procedures. The industry can fully realize the potential of phages as sustainable solutions for agricultural biocontrol and food safety by utilizing AI, innovative production technologies, and a single regulatory framework.

## 6 Conclusion

In the agri-food sector, phages offer a viable and long-term way to fight bacterial infections. Because of their specificity, versatility, and compatibility with contemporary biocontrol techniques, they are handy tools for dealing with issues like food safety and antibiotic resistance. However, effective production procedures that guarantee scalability, regulatory compliance, and product efficacy are necessary to realize their full potential.

This review thoroughly analyzes the upstream and downstream processes necessary for phage production to highlight the crucial steps for optimizing phage production and purity. Despite the optimization of multi-stage bioreactor systems in upstream processing and advances in efficient purification technologies and stable formulations in downstream processing, there is still room for improvement in the scale-up and effective use of more efficient methods in manufacturing processes. Closing this gap is crucial to producing stable, high-quality phage formulations for various uses, including agricultural biocontrol and food safety.

Future developments in phage production should concentrate on using cutting-edge, high-throughput, and scalable technologies, such as continuous processing and combined purification methods, to increase productivity and reduce expenses. Furthermore, using Industry 4.0 approaches, AI and machine learning techniques provide a revolutionary way to streamline QC, optimize bioprocess parameters, and customize phage products to target certain bacteria. A single regulatory framework is also essential to unify production procedures and guarantee safety without sacrificing the efficacy of phage-based therapies.

The phage manufacturing sector has the potential to transform biocontrol tactics in the agri-food business by filling existing gaps and encouraging cooperation between academia, industry, and regulatory agencies. This will open the door to practical and long-lasting ways to defend agricultural practices and food systems from bacterial threats.

## CRediT authorship contribution statement

**Elham Mohammadi:** Writing – review & editing, Writing – original draft, Data curation. **Mohammadreza Rahimian:** Writing – review & editing, Writing – original draft, Visualization, Formal analysis, Data curation. **Bahman Panahi:** Writing – review & editing, Conceptualization.

## Declaration of competing interest

The authors declare that there is not any conflict of interest

## Data Availability

Data will be made available on request.
